# The Role of Nutrition in the Treatment of Sarcopenia in Old Patients: From Restoration of Mitochondrial Activity to Improvement of Muscle Performance, a Systematic Review

**DOI:** 10.3390/nu15173703

**Published:** 2023-08-24

**Authors:** Camille Cochet, Giulia Belloni, Ilaria Buondonno, Francesco Chiara, Patrizia D’Amelio

**Affiliations:** 1 Service of Geriatric Medicine and Geriatric Rehabilitation, Department of Internal Medicine, University of Lausanne Hospital Centre (CHUV), 1011 Lausanne, Switzerland; camilleanais.cochet@gmail.com; 2 Department of Epidemiology and Health Systems, Center for Primary Care and Public Health (Unisanté), University of Lausanne, 1011 Lausanne, Switzerland; 3 Laboratory of Osteobiology and Aging Diseases, Department of Medical Sciences, University of Turin, Via Santena 5 bis, 10124 Turin, Italy; 4 Clinical Biochemistry Laboratory, City of Health and Science University Hospital, 10043 Turin, Italy

**Keywords:** sarcopenia, malnutrition, mitochondrial bioenergetic, redox activity, older adults, BCAA, vitamin D, omega-3 PUFA, geriatrics

## Abstract

Sarcopenia is an age-related disease characterized by loss of muscle strength, mass and performance. Malnutrition contributes to sarcopenia pathogenesis. The aim of this systematic review is to analyze existing evidence on the efficacy of nutritional supplementation on muscle and mitochondrial health among sarcopenic or malnourished older adults. We included randomized controlled trials (RCTs) assessing the effect of branched-chain amino acid (BCAA), vitamin D and/or omega-3 polyunsaturated fatty acid (PUFA) on muscle mass, strength and performance and/or on mitochondrial activity and redox state in older sarcopenic and/or malnourished adults. The literature search was on MEDLINE, Embase and Cochrane Central, restricted to articles published in the last 10 years (2012–2022). Twelve RCTs with a total of 1337 subjects were included. BCAA with vitamin D significantly ameliorates appendicular muscle mass (4 RCTs), hand grip strength (4 RCTs), gait speed (3 RCTs), short physical performance battery (3 RCTs) or chair stand test (3 RCTs) among six out of nine RCTs. BCAA alone (2 RCTs) or PUFA (1 RCT) were not effective in improving muscle health. Mitochondrial function was significantly improved by the administration of BCAA alone (1 RCT) or in association with vitamin D (1 RCT). In conclusion, BCAA in association with vitamin D may be useful in the treatment of sarcopenia and boost mitochondrial bioenergetic and redox activity. PROSPERO CRD42022332288.

## 1. Introduction

Sarcopenia is a condition characterized by loss of muscle strength, mass and performance. Sarcopenia reduces independency, increases risk of falls and hospital admissions, reduces mobility and increases mortality. This disease is frequently associated with aging and plays a major role in the development of frailty syndrome [1]. The worldwide prevalence of sarcopenia in people older than 60 years is estimated between 10% and 27%, the use of different diagnostic criteria [2] contributes to the under-diagnosis and under treatment of this disease. The ancient criteria essentially considered muscle mass reduction, whereas the more recent criteria take into account mainly the reduction of muscle strength and performance; different diagnostic criteria are summarized in Table 1.

The aging of the population will lead to a significant increase in the prevalence of sarcopenia, increasing the associated socio-economic burden [4]. Therefore, an early diagnosis, an adapted and individualized treatment are essential to counteract the spreading of the disease.

The physiopathology of sarcopenia is complex; several lifestyle factors contribute to its development, including malnutrition and physical inactivity [8,9,10] that are frequent amongst older persons [2]. The muscle being a high-energy-demanding tissue, mitochondrial dysfunction [11,12] associated with aging [13], malnutrition [14] and physical inactivity [14] has been proposed as an important contributor to the pathogenesis of the disease [15]. Aging is associated with a decrease in mitochondrial biogenesis and performance; the impairment of mitochondrial function has been proposed as one of the causes of unhealthy aging [13].

Even though healthy mitochondria reduce oxidative stress levels, their respiratory chain generates reactive oxygen species (ROS) within the organelle; the consequent increase in oxidative stress is counteracted by antioxidant enzymes. Although the production of ROS is physiological at a cellular level, if the balance between ROS and the antioxidant system exceeds a certain threshold, oxidative stress increases and mitochondrial DNA is damaged; this damage induces a mitochondrial protein degradation and impairs mitochondrial function [13].

The impairment of mitochondrial function contributes to the increase in oxidative stress and to the development of immune senescence and immune system dysfunction [16]. The immune system dysfunction associated with unhealthy aging causes an increase in chronic, sterile inflammation, named “inflammaging”. This pro-inflammatory status favors muscle catabolism [17,18] and, hence, sarcopenia. The increase in inflammation and the decrease in mitochondrial function at cellular and molecular levels are associated with unhealthy aging at a clinical level and play a paramount role in the development of sarcopenia [15].

Amongst clinical factors associated with sarcopenia, malnutrition plays an important role. Malnutrition is a complex syndrome with multifactorial causes due to both a quantitative and qualitative reduction of dietary intake and an impairment of gut absorption, and it is frequent amongst older adults. This condition has been defined as an involuntary weight loss of 5% in less than 6 months or 10% in more than 6 months, or a BMI of less than 22, in patients over 70 years old, associated with reduced energy intake or acute injury or chronic-related disease [19]. Several screening tools for the diagnosis of malnutrition have been proposed; in older persons, the more frequently used screening tools are the Mini Nutritional Assessment (MNA) or its short form (MNA-SF) [20,21] and the Nutritional Risk Screening Score (NRS) [22,23]. These tools are user-friendly questionnaires. The three questionnaires focus on BMI, reduced food intake, weight loss and the presence of acute and severe illness. Within the MNA and MNA-SF, the presence of cognitive impairment or depression is considered as well as the patient’s mobility. The full form of the MNA also takes into account dietary habits, self-perceived health, medication, living environment, skin wounds, and calf and arm circumference. The decreased intake/absorption of nutrients essential for muscle health, such as protein, amino acids, vitamins and minerals in malnourished subjects contributes to the development of sarcopenia [15,24].

The negative impact of reduced dietary intake of essential amino acid on muscle synthesis [25] in older age is enhanced by the reduced muscular protein synthesis during aging [14,26], this phenomenon has been named “anabolic resistance” of aging. The impairment in mRNA translation and in the activation of the mammalian target of rapamycin (mTOR) pathway, are amongst the underlying causes of this anabolic resistance. Higher protein intake can counteract the anabolic resistance [27]. Indeed, branched-chain amino acids (BCAAs), particularly leucine, isoleucine and valine, have the capacity to activate the mTOR pathway [28], promoting mitochondrial biogenesis, and boosting the PGC 1 alpha pathway which increases oxidative resistance [14,15,29]. Omega-3 PUFA also contributes to the activation of the mTOR pathway, leading to increased protein mitochondrial synthesis in muscle cells [30]. Furthermore, omega-3 PUFA have an anti-inflammatory effect [31] and can decrease oxidative stress [32] thus counteracting the catabolic effect of inflammaging.

Vitamin D plays a role in maintaining muscle health and, in particular, it influences muscle cells differentiation, maturation and growth [33]. Aging is associated with reduced expression of muscle vitamin D receptor and lower vitamin D level, and these factors contribute to the reduction of muscle mass [34]. Furthermore, at the cellular level, some evidence shows that the active form of vitamin D, 1–25 dihydroxyvitamin D3 or calcitriol, increases mitochondrial biogenesis and function [35], and regulates the expression of genes involved in this process in muscles [36].

Given the role of malnutrition in the pathogenesis of sarcopenia, nutritional interventions targeting mitochondrial health and oxidative stress imbalance are emerging as possible strategies to treat this disease.

The aim of this systematic review is to analyze existing evidence on the efficacy of nutritional supplementation on muscle and mitochondrial health among sarcopenic and/or malnourished older adults. This review is divided into two parts: a clinical section focused on the role of nutritional supplementation on muscle strength, mass and performance and a biological section focused on the role of nutritional supplementation on mitochondrial health. 

## 2. Methods

### 2.1. Study Design

We conducted a systematic review according to the Preferred Reporting Items for Systematic Reviews and Meta-Analyses (PRISMA) statement [37], and Testing Methodological Guidance on the Conduct of Narrative Synthesis in Systematic Reviews [38], as well as Synthesis without meta-analysis reporting guidelines [39]. The review protocol is published on the PROSPERO database (registration number CRD42022332288).

### 2.2. Inclusion and Exclusion Criteria

We included randomized controlled trials (RCTs) and meta-analyses which assessed the effect of nutritional supplementation the clinical parameters for the diagnosis of sarcopenia or the mitochondrial activity in older adults (65 years and older) affected by sarcopenia and/or malnutrition. The nutritional supplements analyzed were BCAA and/or vitamin D and/or omega-3 PUFA. 

The research question was formulated by the following PICOS (participants, intervention, comparison, outcomes, study) strategy.

- Participants: Adults aged 65 years and older affected by sarcopenia and/or malnutrition clinically diagnosed. Sarcopenia was defined using recognized diagnostic criteria, such as the European Working Group on Sarcopenia in Older People (EWGSOP-1 [3] and EWGSOP-2 [4]), FNIH [5], International Working Group [6] or Asian working group on sarcopenia (ASIA) [7,40]. The diagnosis of malnutrition or the presence of a malnutrition risk was defined according to a validated tool such as MNA, MNA-SF or NRS score [23].
- Intervention: BCAA and/or omega-3 PUFA and/or vitamin D supplementation, associated or not to physical exercise.
- Comparison: Standard clinical treatment, placebo, or physical exercise without nutritional supplementation.
- Outcomes: Measurement of the parameters of muscle mass and/or muscle strength and/or muscle performance or the mitochondrial activity and/or the oxidative stress.
- Studies: RCT and meta-analysis.

We excluded trials whose participants were affected by sarcopenia due to the following diseases: kidney or hepatic failure, cancer, neuromuscular disease, AIDS, COVID-19, recent surgery or transplants, or severe neurological disorders. We also excluded clinical trials assessing the effect of any kind of protein, (soy, casein, whey proteins or unknown) alone without BCAA, vitamin D or omega-3 PUFA.

### 2.3. Search Strategy

MEDLINE, Embase, Cochrane Central Register of Controlled Trials (CENTRAL) were used for the literature search; the final search was run on 30 July 2022. A re-run was carried out on 9 August 2023 and retrieved two additional papers, that are discussed in the discussion section. The search was restricted to the last ten years of publications (2012–2022), to studies performed in humans and written in English. 

The search string for the clinical part was as follows: (((“Sarcopenia”[MeSH Terms] OR “Malnutrition”[MeSH Terms]) AND “amino acids, branched chain”[MeSH Terms]) OR “fatty acids, omega 3”[MeSH Terms] OR “Vitamin D”[MeSH Terms]) AND ((y_10[Filter]) AND (meta-analysis[Filter] OR randomizedcontrolledtrial[Filter]) AND (humans[Filter]) AND (english[Filter]) AND (aged[Filter] OR 80 andover[Filter])). The search strategy is publicly available at: ((((“Sarcopenia”[Mesh]) OR “Malnutrition”[Mesh]) AND “Amino Acids, Branched-Chain”[Mesh]) OR “Fatty Acids, Omega-3”[Mesh]) OR “Vitamin D”[Mesh]—Search Results—PubMed (nih.gov), and https://www.cochranelibrary.com/advanced-search/search-manager?search=6982594 (accessed on 9 August 2023).

The search string for the biological part was as follows: (((“amino acids, branched chain”[MeSH Terms] OR “fatty acids, omega 3”[MeSH Terms] OR “Vitamin D”[MeSH Terms]) AND “Mitochondria”[MeSH Terms]) OR “Oxidative Stress”[MeSH Terms]) AND ((y_10[Filter]) AND (meta-analysis[Filter] OR randomizedcontrolledtrial[Filter]) AND (humans[Filter]) AND (english[Filter]) AND (80 andover[Filter] OR aged[Filter])). The search strategy is publicly available at: ((((“Amino Acids, Branched-Chain”[Mesh]) OR “Fatty Acids, Omega-3”[Mesh]) OR “Vitamin D”[Mesh]) AND “Mitochondria”[Mesh]) OR “Oxidative Stress”[Mesh]—Search Results—PubMed (nih.gov) and https://www.cochranelibrary.com/web/cochrane/advanced-search/search-manager?search=6982595 (accessed on 9 August 2023).

A second, less restrictive, search strategy for the biological part was as follows: ((‘mitochondria/exp OR ‘mitochondrion’ OR ‘oxidative stress’/exp OR ‘oxidative stress’) AND (‘vitamin d’/exp OR ‘vitamin d’) OR ‘omega 3 fatty acid’/exp OR ‘omega 3 fatty acid’ OR ‘branched chain amino acid’/exp OR ‘branched chain amino acid’) AND [randomized controlled trial]/lim AND ([aged]/lim OR [very elderly]/lim) AND [humans]/lim AND [2012–2022]/py. The search strategy is publicly available at: https://pubmed.ncbi.nlm.nih.gov/?term=%28%28%28%28%22Amino+Acids%2C+Branched-Chain%22%5BMesh%5D%29+OR+%22Fatty+Acids%2C+Omega-3%22%5BMesh%5D%29+OR+%22Vitamin+D%22%5BMesh%5D%29+AND+%22Mitochondria%22%5BMesh%5D%29+OR+%22Oxidative+Stress%22%5BMesh%5D&filter=pubt.clinicaltrial&filter=pubt.randomizedcontrolledtrial&filter=datesearch.y_10&filter=hum_ani.humans&filter=lang.english&filter=age.aged&filter=age.80andover&show_snippets=off&sort=date (accessed on 9 August 2023).

### 2.4. Study Selection 

Two teams of reviewers worked, according to their expertise, on the clinical part (CC and GB) or on the biological part (FC and IB). The reviewers of each team worked independently on the studies retrieved by the search to evaluate the inclusion of each study; discrepancies between the two reviewers were solved through discussion and if necessary, by the third reviewer (PDA for both parts of the review). The Rayyan^®^ tool was used to detect duplicates and abstracts published before 2012 having escaped our filter, and to a systemized article selection process between the authors (available at Rayyan—Intelligent Systematic Review—Rayyan). The bibliography of included articles was screened to detect other eligible RCTs and subjected to the same process as the RCT detected by the search-string strategy.

### 2.5. Data Extraction

From each study, we extracted the following data: authors, year of publication, location, number and characteristic of participants (mean age and gender), diagnosis at baseline (sarcopenia vs. malnutrition), type and duration of intervention, type of control group used, type of physical exercise, inclusion and exclusion criteria, and sample size and measured outcomes. If applicable, information on falls, length of hospital stay and mortality was also recorded. 

### 2.6. Quality Assessment

The risk of bias was assessed by the RoB 2 tool according to Cochrane (https://methods.cochrane.org/risk-bias-2 (accessed on 14 July 2022)). As for the study selection, two reviewers independently evaluated the bias of each study; discrepancies between the reviewers were solved by discussion and consensus or by the third reviewer (PDA, for both parts of the review). 

### 2.7. Data Synthesis and Analysis

The aim of our work was to write a systematic narrative review, without meta-analysis. To compare the different studies, we grouped them according to the type of intervention. Therefore, three subgroups were identified: 1. Vitamin D + BCAA or Whey protein; 2. BCAA alone; 3. Omega-3 PUFA. 

## 3. Results

### 3.1. Study Selection

For the clinical part, we retrieved 4348 abstracts after searching the following databases: Medline (*n* = 1761), Cochrane Central (*n* = 1744) and Embase (*n* = 843). We removed 1585 duplicates and 382 papers were marked as ineligible by the Rayyan tool (abstracts dating before 2012). We identified 17 supplementary records through the bibliographies of the included articles [41,42,43,44,45,46,47,48,49,50,51,52,53,54,55,56,57]. Hence, forty-six full-text articles were reviewed as previously described. After reading the full text article, we excluded twenty-two articles for violation of inclusion criteria. Twelve articles were excluded due to the following reasons: wrong study design (*n* = 3), wrong outcome (*n* = 4), wrong study population (*n* = 1), wrong intervention (*n* = 2), full paper not-available (*n* = 2). Twelve articles were included in the clinical part of the review [45,47,50,57,58,59,60,61,62,63,64,65]. 

For the biological part, with the first search strip, we retrieved 1542 abstracts, after searching the following databases: Medline (*n* = 776), Cochrane Central (*n* = 193) and Embase (*n* = 573). We removed 196 duplicates. A less restrictive search strategy was re-run and retrieved 652 more abstracts. We excluded 16 articles for violation of inclusion criteria and four articles were excluded due to unavailability of the full paper. Hence, two full-text articles were reviewed as previously described. Two articles were included in the biological part of this review and were also eligible for the clinical part [49,57,58,65]. All the included articles were RCTs; no meta-analyses were included.

**Figure 1:**
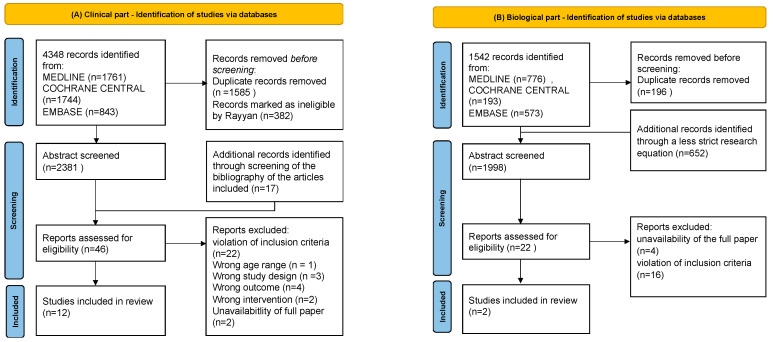
Study’s flow charts. Panel (**A**) shows the flow chart of the clinical part and panel (**B**) shows the flow chart of the biological part.

Figure 1 shows the study’s flowcharts for both the clinical and biological part.

### 3.2. Study Characteristics

Amongst the 12 studies included in the clinical part of the review, seven evaluated nutritional supplementations with BCAA mixture in association with vitamin D [45,50,57,60,61,62,64], one BCAA mixture alone [65], one leucine alone [58], two evaluated whey protein or proteins in association with vitamin D [59,63] and one omega-3 PUFA [47]. All the characteristics of nutritional supplementation are specified in Table 1. Nine studies had a similar timing of intervention between 8 and 12 weeks; one was run for 30 days [64] and two considered a longer intervention period: six months [59] and one year [58]. Amongst the included studies, four included a physical training program; in two [60,61], physical exercise intervention including resistance training with gait and balance training was proposed both in the intervention and the control group. Two studies [45,63] were a four-arm trials comparing nutrition alone, exercise alone, nutrition plus exercise, versus no intervention [63] or health education [45]; exercise consists in resistance training [63] or in resistance and aerobic training [45]. All characteristics of the studies are summarized in Table 2.

### 3.3. Quality Assessment 

**Figure 2:**
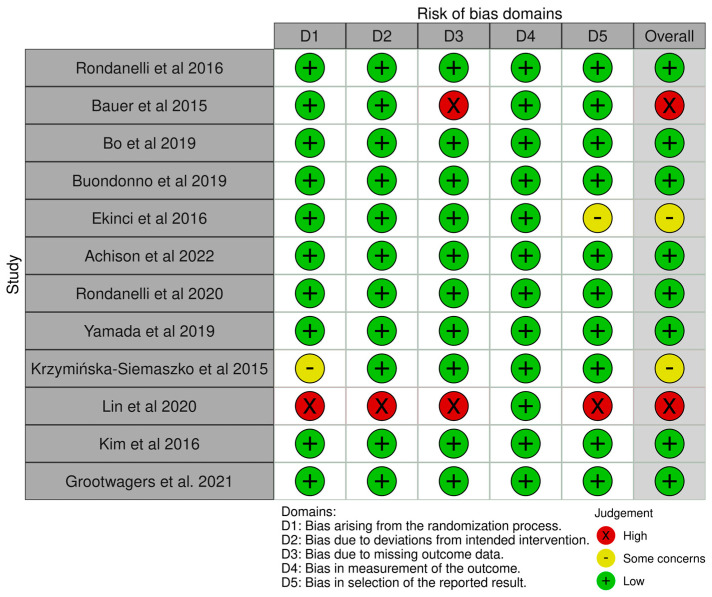
Details of the evaluation of bias risk for each study [45,47,50,57,58,59,60,61,62,63,64,65].

Amongst the included studies, 16.6% raised some concerns or were evaluated at high risk of biases in the randomization process, 8.3% were at high risk from intended interventions, 16.6% were at high risk for missing outcome data, and 25% raised some concerns or were at high risk for reported result. Overall, 25% of the studies have high risk of bias and 8.3% raised some concerns (Figure 2). 

### 3.4. Outcomes of the Included RCT

Five clinical studies out of nine testing vitamin D and whey protein or BCAA supplementation [59,60,61,62,64] found a significant improvement of muscle mass and/or on muscle strength in supplemented patients. Moreover, three studies [57,61,62] out of seven showed a significant effect of this intervention in the amelioration of physical performance; the studies that did not reach statistical significance showed a positive trend in the intervention group. Studies focusing on nutritional supplementation with vitamin D with whey proteins or BCAA administered with physical exercise [45,63] demonstrated a significant effect of the combined intervention on muscle mass, strength or performance. Regarding the biological findings, one study [65] assessing nutritional supplementation with a BCAA mixture showed a significant improvement of mitochondrial bioenergetics and redox activity. The second study showed a positive trend on mitochondrial activity with nutritional supplementation including a vitamin D, whey protein and BCAA mixture. Detailed results of intervention’s studies are summarized in Table 3 for the clinical outcomes and Table 4 for the biological outcomes.

## 4. Discussion

Sarcopenia is a frequent condition amongst older adults and malnutrition plays a causal role on its pathogenesis as well as mitochondrial dysfunction. The use of nutritional supplementation can be effective in improving mitochondrial function and in improving muscle health, thus treating sarcopenia; however, there is no clear recommendation on the type of nutritional supplements useful for treating this disease and ameliorating mitochondrial function. 

Here, we suggest that nutritional supplementation with vitamin D combined with whey proteins or BCAA mixture can be effective in increasing muscle mass, muscle strength or performance, whereas supplementation with BCAA or omega-3 PUFA alone is not. All the studies on vitamin D used cholecalciferol in doses ranging between 100 and 1000 IU per day. Four out of nine studies on this combination demonstrated a significant gain in muscle mass [59,60,61,62] compared to placebo; however, no gain in muscle mass was observed if the control groups received nutritional counseling or a standard nutritional intervention. This result suggests that standard clinical treatment is sufficient to induce an improvement in muscle mass.

Nutritional supplementation with vitamin D combined with whey protein or BCAA may be effective in improving muscle strength [59,60,61,64], regardless of the addition of physical exercise. These findings are particularly relevant in geriatrics as, frequently, older patients are not willing to or are unable to follow an adequate physical activity program [66]; thus, we suggest to treat these patients with the combination of vitamin D and whey protein or BCAA, even without adding physical activity. 

Three out of seven studies [57,61,62] showed a significant improvement of the muscular performance thanks to the nutritional intervention with vitamin D and whey protein or BCAA. The four studies that did not reach statistically significant results showed a trend towards improvement of muscle performance [45,50,59,63]. The included studies differed in the quantity of BCAA and whey proteins administered; the studies showing a significant improvement in muscle performance evaluated doses of total leucine higher than 3 g/day combined with 20 to 40 g of whey proteins/day, administered during the two main meals. Although there is no unique recommendation for the amount of BCAA required to trigger muscular protein synthesis in older patients [67], the literature suggests a dose-dependent correlation between BCAA supplements and muscular protein synthesis induction [68]. It is noteworthy that the only study that did not find significant effects of BCAA on muscle mass, strength or performance [58] administrates leucin without other EAA or protein supplementation, suggesting that, although leucin is necessary to boost muscle health, it is not sufficient and must be administered with other EAA or with whey proteins in order to achieve a clinical benefit. According to Wolfe [69] and Wilkison [70], leucine alone might contribute to an enhanced anabolic response and therefore increase muscle mass, However, all the EAA are needed to induce muscular proteins synthesis [71]. Hence, if only leucine is supplemented, the other needed EAA will be obtained from muscle protein breakdown, blunting the efficacy of leucine. 

Although nutritional supplementation can boost muscular health even without physical exercise, adding aerobic or resistance training will significantly improve muscle mass, strength and performance [45,63]. Physical inactivity increases anabolic resistance [14] and hence contributes to muscle waste and sarcopenia; moreover, physical exercise enhances the effect of BCAA and protein supplementation as muscle protein breakdown during exercise releases EAA that boost protein synthesis [69]. A meta-analysis [72] published after the end of our research, including three RCTs, also included in our review [60,61,62], concludes that supplementation with whey protein, leucine and vitamin D, without physical exercise, increases muscle mass in sarcopenic patients; if nutritional supplementation is combined with physical activity, it also increases muscle strength and performances. Differently from Chang and Choo [72], we included in our systematic review not only supplementation with protein, leucine, and vitamin D in sarcopenic patients but also BCAA and omega-3 PUFA and widen the target population by also including malnourished patients. Despite these differences, our work supports their findings and contributes to generalization to a wider population. 

Thus, we suggest adding appropriate and adapted training to nutritional supplementation whenever possible.

Only two studies evaluated the effect of nutritional supplements on mitochondrial activity: one study focused on BCAA alone [65], the other studied the effect of BCAA plus vitamin D [57]. BCAA were proven to be effective in ameliorating mitochondrial bioenergetics and function even without the addition of vitamin D by Buondonno et al. [65]. These authors showed an increase in the ability of mitochondria to produce ATP and an increased ability to reduce oxidative stress over two months. The authors also evaluated clinical outcomes on muscle mass, strength and performance, showing a positive trend on muscle performance; thus, they hypothesize that the improvement of mitochondrial activity via the mTOR signaling pathway and reduction of ROS may explain the improvement of muscle performance. 

Grootswagers et al. [57] evaluated the effect of nutritional supplementation with BCAA and vitamin D on mitochondrial activity showing that this treatment is effective in improving mitochondrial bioenergetics and function, and that this improvement might explain the amelioration of muscular performance; the authors demonstrated that patients with improved mitochondrial biogenesis had a significant improvement in the gait speed, whereas the others did not.

The results from these two studies suggest that nutritional supplementation may play a role in improving age-associated mitochondrial dysfunction, paving the way for further studies on other conditions associated with unhealthy aging, such as cognitive impairment. In this regard, Buondonno et al. [65] showed an improvement not only in muscular, but also in cognitive performance in old malnourished patients treated with BCAA, this improvement correlated with the increase in ATP production by mitochondria. 

Even if some papers suggest that omega-3 PUFA may be beneficial in sarcopenic patients [30], we cannot draw any conclusions on their efficacy as only one study was eligible for our review [47]. For instance, the results of this study were not significant; these authors tested omega-3 PUFA in addition to vitamin E that was also administered to the control group. After the conclusion of our search, an RCT tested the impact of supplementation with omega-3 PUFA (500 mg), leucine (2.5 g) and Lactobacillus paracasei PS23 in a population of sarcopenic patients [73]. This study demonstrated a significant improvement in muscle mass, strength and performance in patients supplemented, compared to the placebo group. Although this study shows positive results, the researchers used a mixture of supplements; thus, it is not possible to conclude in favor of the supplementation with the sole omega-3 PUFA.

A major limitation for this review is the small sample size of the studies included and the presence of several biases in the study designs that are often not double blind and placebo controlled. Moreover, in this review, we could not evaluate the effect of vitamin D alone. Even though several authors showed that active vitamin D supplementation (calcitriol) significantly improves mitochondrial function, biogenesis and decreases oxidative stress [35,36,74], and, clinically, hypovitaminosis D induces muscle atrophy, clinical trials on vitamin D supplementation were not effective in proving its effectiveness on muscle mass, strength and function [75].

One of the strengths of our review is to focus on BCAA rather than on whey proteins; indeed, even if whey proteins contain BCAA (approximately 11%) [14], their quality, quantity and bioavailability might not be sufficient in order to activate muscle anabolism.

Another significant strength of our review is to include only studies on patients suffering from sarcopenia or malnutrition. This restriction allows us to reduce the heterogeneity between the studies as concerns the targeted population.

To the best of our knowledge, this review is the first to evaluate the impact of targeted nutritional supplementation on both biological and clinical outcomes and to shed light on their relationship. 

## 5. Conclusions

Although there is still room for well-designed studies on the optimal treatment strategy for older malnourished patients, the results of this systematic review suggest that the treatment for these patients is vitamin D supplementation associated with either BCAA or whey proteins together with appropriate aerobic or anaerobic training.

All the studies included in this review used cholecalciferol in doses ranging between 100 and 1000 IU per day, and even at the lower doses [60], the supplementation of vitamin D coupled with BCAA and whey proteins is effective in ameliorating muscle health; hence, we recommend to give cholecalciferol 1000 IU daily in patients at increased risk of vitamin D deficiency according to the recent guidelines from the Society of Clinical and Economical Aspects of Osteoporosis, Osteoarthritis and Musculoskeletal Diseases (ESCEO) working group [76].

As regards the BCAA to be used, a mixture of amino acids including leucin, valine and isoleucine with a 2:1:1 ratio [65,77] or 4:1:1 ratio [45,60] works better than the sole leucine that might be used at doses equal to or higher than 3 g/day [69]. If whey proteins are used, the intake needed to overcome anabolic resistance in older sarcopenic patients is at least 1.2 g/kg/day [78]. These doses are above the internationally recommended dietary allowance of 0.8 g/kg/day [14,15,78] and can cause gastrointestinal problems or be contraindicated in case of kidney failure. These limitations in the use of whey proteins must be considered in the clinical prescription of nutritional supplementation in older patients. 

Regarding the timing of protein intake, we suggest distributing proteins and or BCAA supplements over the three main meals to boost protein synthesis after each meal [14].

We also suggest using BCAA with or without vitamin D to boost mitochondrial activity and potentially ameliorate other chronic conditions associated with aging, such as cognitive impairment.

As concerns omega-3 PUFA, we cannot, based on the results of this review, recommend their use in sarcopenic older adults.

## Figures and Tables

**Table 1:** Different criteria for the diagnosis of sarcopenia.

Criteria	Muscle Mass	Muscle Strength	Muscle Performance	Summary Definition
European Working Group on Sarcopenia in Older People (EWGSOP1, 2010) [3]	2 SD < mean reference value	Grip Strength: <30 kg	Gait Speed < 0.8 m/s.	Sarcopenia: Low muscle mass + low muscle strength OR low performance. Severe sarcopenia: All 3 criteria.
European Working Group on Sarcopenia in Older People (EWGSOP2, 2019) [4]	ASM M < 20 kg; F < 15 kg ASM/height^2^ M < 7.0 kg/m^2^; F < 5.5 kg/m^2^	Grip Strength: M < 27 kg, F < 16 kg Chair stand: >15 s for five rises	Gait Speed < 0.8 m/s SPPB < 8 TUG < 20 s 400 m walk test > 6 min or non-completion	Assess sarcopenia with low muscle strength confirmed sarcopenia with low muscle mass. Class Sarcopenia’s severity with performance. Severe sarcopenia: All 3 criteria.
Foundation for the National Institutes of Health (FNIH, 2014) [5]	ALM/BMI M < 0.789; F < 0.512	Grip Strength: M < 26 kg; F < 16 kg	Gait speed < 0.8 ms	Sarcopenia: Low muscle mass and low muscle strength. Class Sarcopenia’s severity with performance severe sarcopenia: All 3 criteria.
International Working Group (2011) [6]	ALM//height^2^ M < 7.23 kg/m^2^ F < 5.67 kg/m^2^	NA	Gait Speed < 1.0 m/s	Sarcopenia: Low muscle mass and low muscle performance.
Asian Working Group for Sarcopenia (ASIA, 2019) [7]	ASM//height^2^ DXA: M < 7.0 kg/m^2^; F < 5.4 kg/m^2^ or BIA: M < 7.0 kg/m; F < 5.7 kg/m^2^	Grip Strength: M < 28 kg; F < 18 kg	Gait Speed < 1 m/s. 6-metre walk < 1.0 m/s 5-time CST ≥ 12 s SPPB ≤ 9	Sarcopenia: Low muscle mass + low muscle strength OR Low physical performance. Severe sarcopenia: All 3 criteria.

ASM: Appendicular Skeletal Muscle Mass; ALM: Appendicular Lean Mass; BIA: Bioelectrical impedance analysis; CST: chair stand test; F: female; M: male; SD: Standard Deviation; SMM: Skeletal Muscle Mass; SPPB: short physical performance battery; TUG: Timed up and go.

**Table 2:** Characteristics of the RCT included within the clinical and biological part.

Authors Year Location	Number of Participants	Mean Age (Years)	Gender (Women %)	Diagnosis (Used Criteria)	Duration of the Intervention	Type of Intervention	Control	Physical Exercice	Outcomes Measured
BCAA or Whey Protein with Vitamin D									
Rondanelli 2016 [60] Italy	130	80.4	59	sarcopenia (EWGSOP1)	12 weeks	Once daily EAA: Leucine 4 g, Isoleucine 1 g, Valine 1 g, L-Lysine, 1.5 g L-Threonine 1.1 g L-Tryptophane 0.3 g, L-Valine 1.0 g NEAA: DL-Met 0.6 g, L-Cys 0.4 g, L-Phe 0.5 g, L-Tyr 0.5 g, Asp 1.8 g, Ser 0.8 g, Glu 5.2 g, Pro 1.0 g, Gly 0.3 g, Ala 0.8 g, Arg 0.8 g Vitamin D3 312 IU Whey protein 68.9 g	Placebo	Yes (RT, gait and balance training)	MM: Relative skeletal muscle mass; MS: Hand grip
Rondanelli 2020 [61] Italy	140	81	63	sarcopenia (EWGSOP1)	8 weeks	Twice daily Leucine 2.8 g Vitamin D 800 IU Whey proteins 20 g	Placebo	Yes (RT, gait and balance training)	MM: Skeletal muscle index, appendicular muscle mass; MS: Hand grip; MP: Gait speed, chair stand test, TUG, SPPB
Lin 2020 [50] Taiwan	56	73.1	28.6	sarcopenia (ASIA 2019)	12-weeks	Once daily Leucine 1.2 g Vitamin D 120 IU Whey protein 8.5 g +Diet advice	Diet advice; instructed to consume 1.5 g protein/kg/BW/day	No	MM: Appendicular muscle mass index; MS: Hand grip; MP: Gait speed
Bauer 2015 [62] Germany	380	77.7	65.5	sarcopenia (EWGSOP1)	13 weeks	Twice daily Leucine 3 g Vitamin D3 800 IU Whey protein 20 g	Placebo	No	MM: Skeletal muscle index; MS: Hand grip; MP: SPPB
Kim 2016 [45] Japan	139	81.1	NI	sarcopenic obesity	3 months	Once daily EAA: Leucine 1.20 g, lysine HCL 0.50 g, valine 0.33 g, isoleucine 0.32 g, threonine 0.28 g, phenylalanine 0.20 g and other 0.17 g Vitamin D 800 IU Tea catechin 540 mg	Exercise Exercise + Nutrition Health education	Yes (RT and AT)	MM: Skeletal muscle mass index; MS: Hand grip, knee extension strength; MP: Gait speed
Grootswagers 2021 [57] The Netherlands	9 (malnourish)	74.1	20.5	malnutrition (MNA-sf)	12 weeks	Twice daily free BCAA 7 g Vitamin D3 432 IU Urosalic acid 206 mg Whey protein 11 g Casein protein 11 g	Twice daily Oral standard nutritional supplementation (with Vit D3 172 IU)	No	MM: Appendicular muscle mass index; MS: Hand grip, knee extension and flexion; MP: SPPB mRNA expression of mitochondrial activity and biogenesis (PGC1- alpha, AMPK, TFAM, redox activity)
Ekinci 2016 [64] Turkey	62	82.6	100	malnutrition (NRS)	30 days	Twice daily Vitamin D 1000 IU CaHMB 3 g Protein 36 g (unknown origin)	Standard nutrition	No	MM: Calf and arm circumference, triceps skinfold thickness; MS: Hand grip
Bo 2019 [59] China	60	74	55	sarcopenia (ASIA 2014)	6 months	Twice daily Vitamin D 702 UI Whey proteins 22 g	Placebo	No	MM: Relative muscle mass index; MS: Hand grip; MP: Gait speed, TUG, chair stand test
Yamada 2019 [63] Japan	34 (sarcopenic)	No data for sarcopenic patient	No data for sarcopenic patient	sarcopenia and dynapenia (ASIA 2014)	12 weeks	Once daily Vitamin D 800 IU Whey protein 10 g	Exercise Exercise + Nutrition Nothing	Yes (RT)	MM: Appendicular muscle mass, MS: Hand grip, knee extension; MP: Gait speed, chair stand test, one-leg stand test
BCAA alone									
Buondonno 2020 [65] Italy	155	83	72.5	malnutrition (MNA)	2 months	Twice daily EAA:Leucine 1.25 g, Lysine 0.65 g, Isoleucine 0.625 g, Valine 0.625 g, Threonine 0.35 g, Histidine 0.15 g, Phenylalanine 0.01 g, Methionine 0.05 g, Tryptophan 0.02 g, NEAA: Cystine 0.15 g, Tyrosine 0.03 g Vitamin B 6 0.1 mg, Vitamin B1 0.15 mg	Nutritional conseilling	No	MM: Calf and arm circumference; MS: Hand grip; MP: Gait speed, TUG, Tinetti, chair stand test Mitochondrial activity (ATP, electron flux); Mitochondiral biogenesis (COX-1, COX-4, TFAM, NRF-1, MFN-1, MFN-2); redoc activity (TBARs)
Achison 2022 [58] UK	145	78.4	54	sarcopenia (EWGSOP1)	12 months	3 times daily Leucine 2.5 g	Placebo	No	MM: Appendicular muscle mass index; MS: Hand grip, quadriceps strentgh; MP: Gait speed, SPPB, Chair stand test, 6 min walk
Omega-3									
Krzymińska-Siemaszko 2015 [47] Poland	27	75.8	59	sarcopenia (EWGSOP1)	12 weeks	Once daily *n*-3 PUFA 1.3 g with vitamine E	Once daily vitamin E 11 mg	No	MM: Appendicular muscle mass index; MS: Hand grip; MP: Gait speed, TUG

Essential amino acid (EAA): histidine, isoleucine, leucine, lysine, methionine, phenylalanine, threonine, tryptophane, and valine. Non-essential amino acid (NEAA): alanine, arginine, asparagine, aspartic acid, cysteine, glutamic acid, glutamine, glycine, proline, serine, and tyrosine. MM: muscle mass; MS: muscle strength; MP: muscle performance. Resistance training (RT), aerobic training (AT), short physical performance battery (SPPB) which consists of (1) a 4-m usual gait speed walking test, (2) a repeated chair rise test and (3) a balance test. TUG: timed up and go test. COX-1 and COX-4: Cytochrome C oxidase-1 and 4; TFAM: Mitochondrial Transcription Factor A; MFN-: Mitofusin-1; MFN-2: Mitofusin-2; TBARs: Thiobarbituric Acid-Reactive Substances. MNA-sf: Mini Nutritional Assessment Scale Short Form; NRS: nutritional risk score; CaHMB: Calcium β-Hydroxy-β-Methylbutyrate; EWGSOP-1: European Working Group on Sarcopenia in Older People; ASIA: Asian Working Group for Sarcopenia.

**Table 3:** Clinical Outcomes.

Authors Year Location	Overall Risk of Bias	Mucsle Mass	Muscle Strength	Muscle Performance
BCAA or Whey Protein with Vitamin D				
Rondanelli 2016 [60] Italy	Low	RSMM (kg/m^2^) Treatment effect (mean difference): 0.27, 95% CI (0.07–0.47), *p* 0.009	Handgrip strength (kg) Treatment effect (mean difference): 3.68, 95% CI (2.55–4.81), *p* < 0.001	NA
Rondanelli 2020 [61] Italy	Low	SMMI (kg/m^2^/month) Crude between-group difference: 0.40, 95% CI (0.06 to 0.73), *p* 0.023	Handgrip strength (kg/month) Crude between-group difference: 5.45, 95% CI (4.51 to 6.38), *p* < 0.001	4 m gait speed (m/s/month) Crude between-group difference: 0.062, 95% CI (0.043 to 0.082), *p* < 0.001 Chair stand test (s/month) Crude between-group difference: 12.64, 95% CI (10.84 to 14.44), *p* < 0.001 Timed up and go (s/month) Crude between-group difference: 3.71, 95% CI (3.09 to 4.33), *p* < 0.001 SPPB (score/month) crude Between-group difference: 2.27, 95% CI (1.88 to 2.68), *p* < 0.001
Lin 2020 [50] Taiwan	High	AMMI (kg/m^2^) Within group change in the intervention group from baseline (6.1 ± 0.64) to 12 weeks (6.56 ± 0.95), *p* < 0.001 Within group change in the control group from baseline (6.27 ± 0.68) to 12 weeks (6.61 ± 0.76), *p* < 0.001	Handgrip strength (kg) Within group change in the intervention group from baseline (25.3 ± 10.1) to 12 weeks (26.1 ± 7.76), *p* 0.19 Within group change in the control group from baseline (26.3 ± 6.95) to 12 weeks (27.6 ± 7.0), *p* 0.03	Gait speed (m/s) Within group change in the intervention group from baseline (0.98 ± 0.14) to 12 weeks (0.97 ± 0.13) *p* 0.016 Within group change in the control group from baseline (0.98 ± 0.14) to 12 weeks (0.97 ± 0.13), *p* 0.58
Bauer 2015 [62] Germany	High	SMI (kg) Treatment effect (mean difference): 0.17, 95% CI (0.004–0.338), *p* 0.045	Handgrip strength (kg) Treatment effect (mean difference): 0.30, 95% CI (−0.46–1.05), *p* 0.44	Gait speed (m/s) Treatment effect (mean difference): 0.01, 95% CI (−0.02–0.04), *p* 0.46 SPPB (score) Treatment effect (mean difference): 0.11, 95% CI (−0.21–0.42), *p* 0.51 Chair stand test (s) Treatment effect (mean difference): −1.01, 95% CI (−1.77–0.19), *p* 0.018
Kim 2016 [45] Japan	Low	SMI (kg/m^2^) Odds Ratio for Changes compared to HE group: NU: 0.78 (0.25–2.21) EX: 0.83 (0.29–2.41) EXNU: 0.67 (0.23–1.93)	Handgrip and knee extension strength (kg) Odds Ratio for changes compared to HE group: NU: 2.71 (0.96–7.64) EX: 3.72 (1.24–11.17) EXNU: 3.69 (1.28–10.71)	Gait speed (m/s) Odds Ratio for Changes compared to HE group: NU: 1.53 (0.52–4.55) EX: 2.06 (0.67–6.29) EXNU: 3.05 (1.01–9.19)
Grootswagers 2021 [57] The Netherlands	Low	ALMI (kg/m^2^) Treatment*time interaction *p* > 0.05	Non-dominant knee extension (Newton) Mean change in the intervention group: 8 ± 12, *p* 1.000 Mean change in the control group: 38 ± 10, *p* 0.003 Treatment*time interaction *p* 0.058 Dominant knee extension (Newton) Mean change in the intervention group: −2 ± 14, *p* 1.000 Mean change in the control group: 26 ± 21, *p* 1.000 Treatment*time interaction *p* 0.145 Dominant knee flexion (Newton) Mean change in the intervention group: 12 ± 9, *p* 1.000 Mean change in the control group: 23 ± 8, *p* 0.036 Treatment*time interaction *p* 0.351 Handgrip strength (dominant hand, kg) Mean change in the intervention group: 0 ± 1, *p* 1.000 Mean change in the control group: 0 ± 1, *p* 1.000 Treatment*time interaction *p* 0.948	400 m walk test (s) Mean change in the intervention group: −7.4 ± 8.7, *p* 1.000 Mean change in the control group: 17.6 ± 7.8, *p* 0.172 Treatment*time interaction *p* 0.038 4 m walk test (s) Mean change in the intervention group: −0.4 ± 0.1, *p* 0.047 Mean change in the control group: 0.0 ± 0.1, *p* 1.000 Treatment*time interaction *p* 0.048 Chair rise test (s) Mean change in the intervention group: 0.0 ± 0.5, *p* 1.000 Mean change in the control group: −0.3 ± 0.4, *p* 1.000 Treatment*time interaction *p* 0.634 SPPB (score) Mean change in the intervention group: 0.1 ± 0.2, *p* 1.000 Mean change in the control group: 0.3 ± 0.2, *p* 0.523 Treatment*time interaction *p* 0.355
Ekinci 2016 [64] Turkey	Some concerns	Arm circumference (cm) Change in the intervention group from baseline (24.00 ± 2.57) to 30 days (24.84 ± 2.00), *p* 0.001 Change in the control group from baseline (25.30 ± 2.53) to 30 days (24.87 ± 2.62), *p* 0.320 Difference between group *p* 0.969 Calf circumference (cm) Change in the intervention group from baseline (41.13 ± 4.19) to 30 days (40.56 ± 3.78), *p* 0.672 Change in the control group from baseline (41.40 ± 3.04) to 30 days (41.77 ± 3.15), *p* 0.986 Difference between group *p* 0.180 Triceps skinfold thickness (TST) (mm) Change in the intervention group from baseline (12.47 ± 3.89) to 30 days (13.94 ± 3.81), *p* < 0.001 Change in the control group from baseline (13.53 ± 3.01) to 30 days (13.13 ± 3.66), *p* 0.999 Difference between group *p* 0.400	Handgrip strength (kg) Change in the intervention group from baseline (7.13 ± 4.01) to 30 days (8.63 ± 3.83), *p* 0.015 Change in the control group from baseline (5.53 ± 3.42) to 30 days (6.40 ± 3.86), *p* 0.157 Difference between group *p* 0.026	NA
Bo 2019 [59] China	Low	RSMi (kg/m^2^) Treatment effect (mean difference): 0.18, 95% CI (0.01–0.35), *p* 0.040	Handgrip strength (kg) Treatment effect (mean difference): 2.68, 95% CI (0.71–4.65), *p* 0.009	6 m gait speed (walking at usual pace) (m/s) Mean change: Intervention group: 0.14 ± 0.15, *p* < 0.001 Control group: 0.08 ± 0.24, *p* 0.074 Treatment effect (mean difference): 0.05, 95% CI (−0.06 to 0.15), *p* 0.402 Timed up and go (s) Mean change: Intervention group: -1.36 ± 2.43, *p* < 0.001 Control group: −0.68 ± 3.29, *p* 0.267 Treatment effect (mean difference): −0.67, 95% CI (−2.20 to 0.86), *p* 0.383 Chair stand test (s) Mean change: Intervention group: −2.79 ± 3.73, *p* 0.005 Control group: −1.21 ± 6.28, *p* 0.507 Treatment effect (mean difference): −1.84, 95% CI (−4.53 to 0.85), *p* 0.176
Yamada 2019 [63] Japan	Low	Appendicular muscle mass (kg) Median change from baseline (IQR range): EXNU: 0.51 (0.04–1.24) EX: −0.30 (−0.64 to 1.07) NU: 0.11 (−0.29 to 0.40) Control group: −0.72 (−1.88 to 0.01) Differences between EXNU and control group *p* 0.02	Maximal isometric knee extension strength (Newton) Median change from baseline (IQR range): EXNU: 39.20 (31.36–45.20) EX: 1.84 (−6.13 to 10.05) NU: −4.41 (−10.17 to 10.41) Control group: 3.92 (−7.60 to 9.43) Differences between EXNU group and control group *p* 0.46 Handgrip strength (kg) Median change from baseline (IQR range): EXNU: 1.70 (−0.20 to 2.70) EX: −0.05 (−2.45 to 1.08) NU: −0.40 (−1.90 to 0.95) Control group: −0.67 (−3.18 to 0.73) Differences between EXNU group and control group *p* 0.07	5 m maximum walking time (s) Median change from baseline (IQR range): EXNU: −0.82 (−1.28 to −0.52) EX: −0.57 (−2.05–0.47) NU: −0.04 (−0.88 to 0.41) Control group: 0.44 (−0.26 to 1.11) Differences between EXNU group and control group *p* 0.01 Five-repetition chair stand test (s) Median change from baseline (IQR range): EXNU: −1.15 (−2.48 to −0.26) EX: −0.27 (−1.92 to 0.00) NU: −0.41 (−1.46 to 0.06) Control group: −0.37 (−1.68 to 0.77) Differences between EXNU group and control group *p* 0.47
BCAA alone				
Buondonno 2020 [65] Italy	Low	Calf circumference (cm) Mean in intervention group at baseline 30.4 ± 0.35, 95% CI (−1.14 to −0.04) and 2 months 31.3 ± 0.39, 95% CI (−1.45 to −0.36) Mean in control group at baseline 30.7 ± 0.43, 95% CI (−1.08 to 0.02) and 2 months 31.19 ± 0.39, 95% CI (−1.07 to 0.03) Time difference *p* 0.0004, treatment difference *p* 0.8560, interaction difference *p* 0.4521 Arm circumference (cm) Mean in intervention group at baseline 22.7 ± 0.36, 95% CI (−0.75 to 0.23) and 2 months 23.3 ± 0.38, 95% CI (−1.03 to −0.04) Mean in control group at baseline 23.0 ± 0.40, 95% CI (−0.83 to 0.15) and 2 months 23.4 ± 0.44, 95% CI (−0.88 to −0.10) Time difference *p* 0.0045, treatment difference *p* 0.6754, interaction difference *p* 0.7351	Handgrip strength (kg) Mean in intervention group at baseline 17.9 ± 1.0, 95% CI (−2.01 to 0.47) and 2 months 18.3 ± 1.0, 95% CI (−2.4 to 0.10) Mean in control group at baseline 17.9 ± 1.0, 95% CI (−1.84 to 0.65) and 2 months 19.1 ± 1.0, 95% CI (−1.59 to 0.90) Time mean difference *p* 0.0474, treatment mean difference *p* 0.7796, Interaction mean difference *p* 0.5231	4 m gait speed (s) Mean in intervention group at baseline 8.2 ± 0.6, 95% CI (−0.3 to 1.7) and 2 months 7.2 ± 0.6, 95% CI (0.04 to 2.0) Mean in control group at baseline 9.8 ± 0.7, 95% CI (0.4 to 2.3) and 2 months 8.0 ± 0.7, 95% CI (0.8 to 2.8) Time mean difference *p* < 0.001, treatment mean difference: *p* 0.1685, Interaction mean difference *p* 0.3955 Timed up and go (s) Mean in intervention group at baseline 19.8 ± 2.14, 95% CI (1.5 to 7.6) and 2 months 15.1 ± 1.1, 95% CI (1.6 to 7.8) Mean in control group at baseline 20.5 ± 1.5, 95% CI (−1.2 to 4.9) and 2 months 17.7 ± 1.7, 95% CI (−0.3 to 5.9) Time mean difference *p* 0.0001, treatment mean difference) *p* 0.2780, interaction mean difference *p* 0.3215 30-s Chair to stand test (s) Mean in intervention group at baseline 6.8 ± 0.5, 95% CI (−2.6 to −0.7) and 2 months 8.5 ± 0.7, 95% CI (−2.7 to −0.7) Mean in control group at baseline 6.0 ± 0.5, 95% CI (−2.4 to −0.5) and 2 months 8.1 ± 0.6, 95% CI (−3.0 to −1.1) Time mean difference *p* < 0.0001, treatment mean difference *p* 0.3328, Interaction mean difference *p* 0.5810 Balance test (Tinetti) Mean in intervention group at baseline 20.4 ± 0.8, 95% CI (−2.1 to −0.1) and 2 months 22.2 ± 0.7, 95% CI (−2.8–−0.8) Mean in control group at baseline 18.3± 0.8, 95% CI (−3.2 to −1.1) and 2 months 20.7 ± 0.9, 95% CI (−3.4 to −1.4) Time mean difference < 0.0001, treatment mean difference: *p* 0.1503, interaction mean difference *p* 0.2076
Achison 2022 [58] UK	Low	RSMI (kg/m^2^) Between-group difference over 12 months follow up: −0.3, 95% CI (−1.0, 0.4), *p* 0.47	Handgrip Strength (kg) Between-group difference over 12 months follow up: −0.3, 95% CI (−1.2, 0.7), *p* 0.55 Quadriceps strength (kg) Between-group difference over 12 months follow up: −1.0, 95% CI (−4.4, 2.4), *p* 0.55	4-m gait speed (m/s): Between-group difference over 12-month follow-up: 0.01, 95% CI (−0.18, 0.19), *p* 0.96 Six min walk (m): Between-group difference over 12-month follow-up: 17, 95% CI (−25, 59), *p* 0.43 SPPB (score) Between-group difference over 12-month follow-up: 0.1, 95% CI (−1.0, 1.1), *p* 0.90 Chair stand test (s) Between-group difference over 12-month follow-up: −3.1, 95% CI (−9.5, 3.3), *p* 0.34
Omega-3				
Krzymińska-Siemaszko 2015 [47] Poland	High	ALMI (kg/m^2^) Change in the intervention group from the baseline: 0.00 ± 0.30 Change in the control group from the baseline: 0.03 ± 0.36 Between-group difference *p* 0.53	Handgrip strength (kg) Change in the intervention group from the baseline: 0.68 ± 1.43 Change in the control group from the baseline: 0.54 ± 2.77 Between-group difference *p* 0.12	4-m walking test (s) Change in the intervention group from the baseline: 0.11 ± 0.26 Change in the control group from the baseline: 0.09 ± 0.13 Between-group difference *p* 0.06 Timed up and go (s) Change in the intervention group from the baseline: 0.05 ± 1.50 Change in the control group from the baseline: 0.42 ± 1.18 Between-group difference *p* 0.11

Results showing an efficacy of the nutritional intervention as compared to placebo are highlighted in shadow. Efficacity of treatment are reported for all the studies RSMM: Relative Skeletal Muscle Mass; SMI: skeletal muscle index; AMS: Appendicular Muscle Mass; AMMI; Appendicular Muscle Mass Index; ALMI: Appendicular Lean Muscle Index; RSMI: relative muscle mass index; SPPB: Short Performance Physical Battery; EXNU: exercise in addition to nutrition; EX: exercise alone; NU: nutrition alone; HE: health education, CI: confidence interval.

**Table 4:** Biological outcomes. Results showing an efficacy of the nutritional intervention as compared to placebo are highlighted in shadow. Efficacity of the treatment is reported for all the studies.

Authors Year Location	Overall Risk of Bias	Mitochondrial Bioenergetics	Mitochondrial Dynamics	Redox Activity
BCAA and Whey Protein with Vitamin D				
Grootswagers 2021 [57] The Netherlands	Low	NA	mRNA PGC-1 alpha (Peroxisome proliferator-activated receptor-γ coactivator-1α) Fold change expression: Intervention group: 4.7 +/− 1.8; Control group: 2.2 +/− 0.6 Between treatment difference *p* 0.685 AMPK (5′adenosine monophosphate-activated protein kinase) Within-treatment difference baseline vs. 12 weeks in intervention group *p* 0.031 Within-treatment difference baseline vs. 12 weeks in control group *p* 0.125 TFAM (Mitochondrial Transcription Factor A) Between treatment difference *p* 0.603	NA
BCAA alone				
Buondonno 2020 [65] Italy	Low	ATP Mean in intervention group at baseline:1.0 ± 0.0, 95% CI (−0.45 to −0.15) and 2 months:1.43 ± 0.10, 95% CI (−0.58 to −0.28) Mean in control group at baseline 1.0 ± 0.0, 95% CI (−0.13 to 0.17) and 2 months 0.99 ± 0.02, 95% CI (−0.14 to 0.16) Time difference *p* 0.0001, Treatment difference *p* 0.0005, Interaction difference *p* 0.0001 Electron flux Mean in intervention group at baseline 1.0 ± 0.0, 95% CI (−0.38 to −0.13) and 2 months 1.50 ± 0.09, 95% CI (−0.62 to −0.38) Mean in control group at baseline 1.0 ± 0.0, 95% CI (−0.13 to 0.13) and 2 months 1.01 ± 0.04, 95% CI (−0.14 to 0.12) Time difference *p* < 0.0001, Treatment difference *p* < 0.0001, Interaction difference *p* < 0.0001	COX-1 (Cytochrome C oxidase -1) Mean in intervention group at baseline 1.0 ± 0.0, 95% CI (−23.8 to −1.9) and 2 months 7.3 ± 3.6, 95% CI (−17.3 to 4.7); Mean in control group at baseline 1.0 ± 0.0, 95% CI (−11.4 to 10.6) and 2 months 3.7 ± 1.2, 95% CI (−13.6 to 8.3) Time difference *p* 0.1155, Treatment difference *p* 0.1967, Interaction difference *p* 0.1409 COX-4 (Cytochrome C oxidase-4) Mean in intervention group at baseline 1.0 ± 0.0, 95% CI (−2.3 to −0.10) and 2 months: 1.8 ± 0.5, 95% CI (−1.9 to 0.3); Mean in control group at baseline 1.0 ± 0.0, 95% CI (−1.39 to 0.76) and 2 months 1.3 ± 0.19, 95% CI (−1.4 to 0.73) Time difference *p* 0.0459, Treatment difference *p* 0.2373, Interaction difference *p* 0.3786 TFAM (Mitochondrial Transcription Factor A) Mean in intervention group at baseline 1.0 ± 0.0, 95% CI (−6.9 to −0.6) and 2 months 4.2 ± 1.15, 95% CI (−6.2 to 0.1); Mean in control group at baseline 1.0 ± 0.0, 95% CI (−3.8 to 2.5) and 2 months: 3.0 ± 1.5, 95% CI (−5.1 to 1.2) Time difference *p* 0.0178, Treatment difference *p* 0.0932, Interaction difference *p* 0.2235 NRF-1 (Nuclear Respiratory Factor-1) Mean in intervention group at baseline 1.0 ± 0.0, 95% CI (−20.2 to 3.5) and 2 months 11.6 ± 9.5, 95% CI (−22.4 to 1.3); Mean in control group at baseline 1.0 ± 0.0, 95% CI (−13.5 to 10.2) and 2 months:3.6 ± 1.2, 95% CI (−14.4 to 9.3) Time difference 0.6599, Treatment difference *p* 0.3507, Interaction difference *p* 0.2055 MFN-1 (Mitofusin-1) Mean in intervention group at baseline 1.0 ± 0.0, 95% CI (−22.4 to −2.1) and 2 months 10.1 ± 6.1, 95% CI (−19.3 to 1.0); Mean in control group at baseline 1.0 ± 0.0, 95% CI (−10.8 to 9.4) and 2 months 1.8 ± 0.3, 95% CI (−11.0 to 9.3) Time difference 0.0746, Treatment difference *p* 0.1648, Interaction difference *p* 0.1320 MFN-2 (Mitofusin-2) Mean in intervention group at baseline 1.0 ± 0.0 95% CI (−11.6 to −1.1) and 2 months 3.9 ± 1.6, 95% CI (−8.2 to 2.3); Mean in control group at baseline 1.0 ± 0.0, 95% CI (−6.0 to 4.5) and 2 months 2.4 ± 0.5, 95% CI (−6.6 to 3.9) Time difference *p* 0.0772, Treatment difference *p* 0.2046, Interaction difference *p* 0.1810	Thiobarbituric Acid Reactiv Substances (TBARs) mcg/M Mean in intervention group at baseline 2.3 ± 0.4, 95% CI (−2.8 to 1.2) and 2 months 3.2 ± 0.70, 95% CI (−3.1 to 0.85) Mean in control group at baseline 4.1 ± 0.7, 95% CI (−3.02 to 0.97) and 2 months 6.7 ± 1.3, 95% CI (−5.64 to −1.64) Time difference *p* 0.0007, Treatment difference *p* 0.0289, Interaction difference *p* 0.0332

Results showing an efficacy of the nutritional intervention as compared to placebo are highlighted in shadow. Efficacity of treatment are reported for all the studies.

## Data Availability

All the data are available to the public and presented in the manuscript.

## References

[1] Cattaneo F., Buondonno I., Cravero D., Sassi F., D’Amelio P. (2022). Musculoskeletal Diseases Role in the Frailty Syndrome: A Case–Control Study. Int. J. Environ. Res. Public Health.

[2] Petermann-Rocha F., Balntzi V., Gray S.R., Lara J., Ho F.K., Pell J.P., Celis-Morales C. (2022). Global Prevalence of Sarcopenia and Severe Sarcopenia: A Systematic Review and Meta-Analysis. J. Cachexia. Sarcopenia Muscle.

[3] Cruz-Jentoft A.J., Baeyens J.P., Bauer J.M., Boirie Y., Cederholm T., Landi F., Martin F.C., Michel J.P., Rolland Y., Schneider S.M. (2010). Sarcopenia: European Consensus on Definition and Diagnosis. Age Ageing.

[4] Cruz-Jentoft A.J., Bahat G., Bauer J., Boirie Y., Bruyère O., Cederholm T., Cooper C., Landi F., Rolland Y., Sayer A.A. (2019). Sarcopenia: Revised European Consensus on Definition and Diagnosis. Age Ageing.

[5] Studenski S.A., Peters K.W., Alley D.E., Cawthon P.M., McLean R.R., Harris T.B., Ferrucci L., Guralnik J.M., Fragala M.S., Kenny A.M. (2014). The FNIH Sarcopenia Project: Rationale, Study Description, Conference Recommendations, and Final Estimates. J. Gerontol. Ser. A Biol. Sci. Med. Sci..

[6] Fielding R.A., Vellas B., Evans W.J., Bhasin S., Morley J.E., Newman A.B., Abellan van Kan G., Andrieu S., Bauer J., Breuille D. (2011). Sarcopenia: An Undiagnosed Condition in Older Adults. Current Consensus Definition: Prevalence, Etiology, and Consequences. International Working Group on Sarcopenia. J. Am. Med. Dir. Assoc..

[7] Chen L.K., Woo J., Assantachai P., Auyeung T.W., Chou M.Y., Iijima K., Jang H.C., Kang L., Kim M., Kim S. (2020). Asian Working Group for Sarcopenia: 2019 Consensus Update on Sarcopenia Diagnosis and Treatment. J. Am. Med. Dir. Assoc..

[8] Rom O., Kaisari S., Aizenbud D., Reznick A.Z. (2012). Lifestyle and Sarcopenia—Etiology, Prevention and Treatment. Rambam Maimonides Med. J..

[9] Marzetti E., Calvani R., Tosato M., Cesari M., Di Bari M., Cherubini A., Broccatelli M., Savera G., D’Elia M., Pahor M. (2017). Physical Activity and Exercise as Countermeasures to Physical Frailty and Sarcopenia. Aging Clin. Exp. Res..

[10] Calvani R., Picca A., Coelho-Júnior H.J., Tosato M., Marzetti E., Landi F. (2023). Diet for the Prevention and Management of Sarcopenia. Metabolism.

[11] Alizadeh Pahlavani H., Laher I., Knechtle B., Zouhal H. (2022). Exercise and Mitochondrial Mechanisms in Patients with Sarcopenia. Front. Physiol..

[12] Hood D.A., Memme J.M., Oliveira A.N., Triolo M. (2019). Maintenance of Skeletal Muscle Mitochondria in Health, Exercise, and Aging. Annu. Rev. Physiol..

[13] Cedikova M., Pitule P., Kripnerova M., Markova M., Kuncová J. (2016). Multiple Roles of Mitochondria in Aging Processes. Physiol. Res..

[14] Moore D.R. (2014). Keeping Older Muscle “Young” through Dietary Protein and Physical Activity. Adv. Nutr..

[15] Romani M., Berger M.M., D’amelio P. (2022). From the Bench to the Bedside: Branched Amino Acid and Micronutrient Strategies to Improve Mitochondrial Dysfunction Leading to Sarcopenia. Nutrients.

[16] Buondonno I., Sassi F., Cattaneo F., D’Amelio P. (2023). Association between Immunosenescence, Mitochondrial Dysfunction and Frailty Syndrome in Older Adults. Cells.

[17] Prado C.M., Landi F., Chew S.T.H., Atherton P.J., Molinger J., Ruck T., Gonzalez M.C. (2022). Advances in Muscle Health and Nutrition: A Toolkit for Healthcare Professionals. Clin. Nutr..

[18] Nelke C., Dziewas R., Minnerup J., Meuth S.G., Ruck T. (2019). Skeletal Muscle as Potential Central Link between Sarcopenia and Immune Senescence. EBioMedicine.

[19] Cederholm T., Jensen G.L., Correia M.I.T.D., Gonzalez M.C., Fukushima R., Higashiguchi T., Baptista G., Barazzoni R., Blaauw R., Coats A. (2019). GLIM Criteria for the Diagnosis of Malnutrition—A Consensus Report from the Global Clinical Nutrition Community. Clin. Nutr..

[20] Garry P.J., Vellas B.J. (1999). Practical and Validated Use of the Mini Nutritional Assessment in Geriatric Evaluation. Nutr. Clin. Care.

[21] Rubenstein L.Z., Harker J.O., Salvà A., Guigoz Y., Vellas B. (2001). Screening for Undernutrition in Geriatric Practice: Developing the Short-Form Mini-Nutritional Assessment (MNA-SF). J. Gerontol. Ser. A Biol. Sci. Med. Sci..

[22] Kondrup J., Ramussen H.H., Hamberg O., Stanga Z., Camilo M., Richardson R., Elia M., Allison S., Meier R., Plauth M. (2003). Nutritional Risk Screening (NRS 2002): A New Method Based on an Analysis of Controlled Clinical Trials. Clin. Nutr..

[23] Cederholm T., Bosaeus I., Barazzoni R., Bauer J., Van Gossum A., Klek S., Muscaritoli M., Nyulasi I., Ockenga J., Schneider S.M. (2015). Diagnostic Criteria for Malnutrition—An ESPEN Consensus Statement. Clin. Nutr..

[24] Norman K., Haß U., Pirlich M. (2021). Malnutrition in Older Adults—Recent Advances and Remaining Challenges. Nutrients.

[25] Atherton P.J., Smith K., Etheridge T., Rankin D., Rennie M.J. (2010). Distinct Anabolic Signalling Responses to Amino Acids in C2C12 Skeletal Muscle Cells. Amino Acids.

[26] Rolland Y., Dupuy C., Abellan van Kan G., Gillette S., Vellas B. (2011). Treatment Strategies for Sarcopenia and Frailty. Med. Clin. N. Am..

[27] Moore D.R., Churchward-Venne T.A., Witard O., Breen L., Burd N.A., Tipton K.D., Phillips S.M. (2015). Protein Ingestion to Stimulate Myofibrillar Protein Synthesis Requires Greater Relative Protein Intakes in Healthy Older versus Younger Men. J. Gerontol. Ser. A Biol. Sci. Med. Sci..

[28] Le Couteur D.G., Solon-Biet S.M., Cogger V.C., Ribeiro R., de Cabo R., Raubenheimer D., Cooney G.J., Simpson S.J. (2020). Branched Chain Amino Acids, Aging and Age-Related Health. Ageing Res. Rev..

[29] D’Antona G., Ragni M., Cardile A., Tedesco L., Dossena M., Bruttini F., Caliaro F., Corsetti G., Bottinelli R., Carruba M.O. (2010). Branched-Chain Amino Acid Supplementation Promotes Survival and Supports Cardiac and Skeletal Muscle Mitochondrial Biogenesis in Middle-Aged Mice. Cell Metab..

[30] Dupont J., Dedeyne L., Dalle S., Koppo K., Gielen E. (2019). The Role of Omega-3 in the Prevention and Treatment of Sarcopenia. Aging Clin. Exp. Res..

[31] Domingues-Faria C., Vasson M.P., Goncalves-Mendes N., Boirie Y., Walrand S. (2016). Skeletal Muscle Regeneration and Impact of Aging and Nutrition. Ageing Res. Rev..

[32] Lalia A.Z., Dasari S., Robinson M.M., Abid H., Morse D.M., Klaus K.A., Lanza I.R. (2017). Influence of Omega-3 Fatty Acids on Skeletal Muscle Protein Metabolism and Mitochondrial Bioenergetics in Older Adults. Aging.

[33] D’amelio P., Quacquarelli L. (2020). Hypovitaminosis d and Aging: Is There a Role in Muscle and Brain Health?. Nutrients.

[34] Bischoff-Ferrari H.A., Borchers M., Gudat F., Dürmüller U., Stähelin H.B., Dick W. (2004). Vitamin D Receptor Expression in Human Muscle Tissue Decreases with Age. J. Bone Miner. Res..

[35] Ryan Z.C., Craig T.A., Folmes C.D., Wang X., Lanza I.R., Schaible N.S., Salisbury J.L., Nair K.S., Terzic A., Sieck G.C. (2016). 1α,25-Dihydroxyvitamin D3 Regulates Mitochondrial Oxygen Consumption and Dynamics in Human Skeletal Muscle Cells. J. Biol. Chem..

[36] Salles J., Chanet A., Guillet C., Vaes A.M., Brouwer-Brolsma E.M., Rocher C., Giraudet C., Patrac V., Meugnier E., Montaurier C. (2022). Vitamin D Status Modulates Mitochondrial Oxidative Capacities in Skeletal Muscle: Role in Sarcopenia. Commun. Biol..

[37] Page M.J., McKenzie J.E., Bossuyt P.M., Boutron I., Hoffmann T.C., Mulrow C.D., Shamseer L., Tetzlaff J.M., Akl E.A., Brennan S.E. (2021). The PRISMA 2020 Statement: An Updated Guideline for Reporting Systematic Reviews. BMJ.

[38] Rodgers M., Sowden A., Petticrew M., Arai L., Roberts H., Britten N., Popay J. (2009). Testing Methodological Guidance on the Conduct of Narrative Synthesis in Systematic Reviews: Effectiveness of Interventions to Promote Smoke Alarm Ownership and Function. Evaluation.

[39] Campbell M., McKenzie J.E., Sowden A., Katikireddi S.V., Brennan S.E., Ellis S., Hartmann-Boyce J., Ryan R., Shepperd S., Thomas J. (2020). Synthesis without Meta-Analysis (SWiM) in Systematic Reviews: Reporting Guideline. BMJ.

[40] Chen L.K., Liu L.K., Woo J., Assantachai P., Auyeung T.W., Bahyah K.S., Chou M.Y., Chen L.Y., Hsu P.S., Krairit O. (2014). Sarcopenia in Asia: Consensus Report of the Asian Working Group for Sarcopenia. J. Am. Med. Dir. Assoc..

[41] Amasene M., Cadenas-Sanchez C., Echeverria I., Sanz B., Alonso C., Tobalina I., Irazusta J., Labayen I., Besga A. (2022). Effects of Resistance Training Intervention along with Leucine-Enriched Whey Protein Supplementation on Sarcopenia and Frailty in Post-Hospitalized Older Adults: Preliminary Findings of a Randomized Controlled Trial. J. Clin. Med..

[42] Cheng H., Kong J., Underwood C., Petocz P., Hirani V., Dawson B., O’Leary F. (2018). Systematic Review and Meta-Analysis of the Effect of Protein and Amino Acid Supplements in Older Adults with Acute or Chronic Conditions. Br. J. Nutr..

[43] Guo Y., Fu X., Hu Q., Chen L., Zuo H. (2022). The Effect of Leucine Supplementation on Sarcopenia-Related Measures in Older Adults: A Systematic Review and Meta-Analysis of 17 Randomized Controlled Trials. Front. Nutr..

[44] Ispoglou T., White H., Preston T., McElhone S., McKenna J., Hind K. (2016). Double-Blind, Placebo-Controlled Pilot Trial of L-Leucine-Enriched Amino-Acid Mixtures on Body Composition and Physical Performance in Men and Women Aged 65–75 Years. Eur. J. Clin. Nutr..

[45] Kim H., Kim M., Kojima N., Fujino K., Hosoi E., Kobayashi H., Somekawa S., Niki Y., Yamashiro Y., Yoshida H. (2016). Exercise and Nutritional Supplementation on Community-Dwelling Elderly Japanese Women with Sarcopenic Obesity: A Randomized Controlled Trial. J. Am. Med. Dir. Assoc..

[46] Kim H.K., Suzuki T., Saito K., Yoshida H., Kobayashi H., Kato H., Katayama M. (2012). Effects of Exercise and Amino Acid Supplementation on Body Composition and Physical Function in Community-Dwelling Elderly Japanese Sarcopenic Women: A Randomized Controlled Trial. J. Am. Geriatr. Soc..

[47] Krzymińska-Siemaszko R., Czepulis N., Lewandowicz M., Zasadzka E., Suwalska A., Witowski J., Wieczorowska-Tobis K. (2015). The Effect of a 12-Week Omega-3 Supplementation on Body Composition, Muscle Strength and Physical Performance in Elderly Individuals with Decreased Muscle Mass. Int. J. Environ. Res. Public Health.

[48] Kemmler W., Grimm A., Bebenek M., Kohl M., von Stengel S. (2018). Effects of Combined Whole-Body Electromyostimulation and Protein Supplementation on Local and Overall Muscle/Fat Distribution in Older Men with Sarcopenic Obesity: The Randomized Controlled Franconia Sarcopenic Obesity (FranSO) Study. Calcif. Tissue Int..

[49] Martínez-Arnau F.M., Fonfría-Vivas R., Buigues C., Castillo Y., Molina P., Hoogland A.J., van Doesburg F., Pruimboom L., Fernández-Garrido J., Cauli O. (2020). Effects of Leucine Administration in Sarcopenia: A Randomized and Placebo-Controlled Clinical Trial. Nutrients.

[50] Lin C.C., Shih M.H., Chen C.D., Yeh S.L. (2021). Effects of Adequate Dietary Protein with Whey Protein, Leucine, and Vitamin D Supplementation on Sarcopenia in Older Adults: An Open-Label, Parallel-Group Study. Clin. Nutr..

[51] Mathieu L., Maltais J.P., Ladouceur A.I.J.D. (2016). Effect of Specific Resistance Training. J. Strength Cond. Res..

[52] Murphy C.H., Flanagan E.M., De Vito G., Susta D., Mitchelson K.A.J., De Marco Castro E., Senden J.M.G., Goessens J.P.B., Mikłosz A., Chabowski A. (2021). Does Supplementation with Leucine-Enriched Protein Alone and in Combination with Fish-Oil-Derived n-3 PUFA Affect Muscle Mass, Strength, Physical Performance, and Muscle Protein Synthesis in Well-Nourished Older Adults? A Randomized, Double-Blind, Placebo. Am. J. Clin. Nutr..

[53] Stow R., Ives N., Smith C., Rick C., Rushton A. (2015). A Cluster Randomised Feasibility Trial Evaluating Nutritional Interventions in the Treatment of Malnutrition in Care Home Adult Residents. Trials.

[54] Takeuchi I., Yoshimura Y., Shimazu S., Jeong S., Yamaga M., Koga H. (2019). Effects of Branched-Chain Amino Acids and Vitamin D Supplementation on Physical Function, Muscle Mass and Strength, and Nutritional Status in Sarcopenic Older Adults Undergoing Hospital-Based Rehabilitation: A Multicenter Randomized Controlled Trial. Geriatr. Gerontol. Int..

[55] Dimori S., Leoni G., Fior L., Gasparotto F. (2018). Clinical Nutrition and Physical Rehabilitation in a Long-Term Care Setting: Preliminary Observations in Sarcopenic Older Patients. Aging Clin. Exp. Res..

[56] Englund D.A., Kirn D.R., Koochek A., Zhu H., Travison T.G., Reid K.F., Von Berens Å., Melin M., Cederholm T., Gustafsson T. (2018). Nutritional Supplementation with Physical Activity Improves Muscle Composition in Mobility-Limited Older Adults, the VIVE2 Study: A Randomized, Double-Blind, Placebo-Controlled Trial. J. Gerontol. Ser. A Biol. Sci. Med. Sci..

[57] Grootswagers P., Smeets E., Oteng A.B., de Groot L. (2021). A Novel Oral Nutritional Supplement Improves Gait Speed and Mitochondrial Functioning Compared to Standard Care in Older Adults with (or at Risk of) Undernutrition: Results from a Randomized Controlled Trial. Aging.

[58] Achison M., Adamson S., Akpan A., Aspray T., Avenell A., Band M.M., Bashir T., Burton L.A., Cvoro V., Donnan P.T. (2022). Effect of Perindopril or Leucine on Physical Performance in Older People with Sarcopenia: The LACE Randomized Controlled Trial. J. Cachexia. Sarcopenia Muscle.

[59] Bo Y., Liu C., Ji Z., Yang R., An Q., Zhang X., You J., Duan D., Sun Y., Zhu Y. (2019). A High Whey Protein, Vitamin D and E Supplement Preserves Muscle Mass, Strength, and Quality of Life in Sarcopenic Older Adults: A Double-Blind Randomized Controlled Trial. Clin. Nutr..

[60] Rondanelli M., Klersy C., Terracol G., Talluri J., Maugeri R., Guido D., Faliva M.A., Solerte B.S., Fioravanti M., Lukaski H. (2016). Whey Protein, Amino Acids, and Vitamin D Supplementation with Physical Activity Increases Fat-Free Mass and Strength, Functionality, and Quality of Life and Decreases Inflammation in Sarcopenic Elderly. Am. J. Clin. Nutr..

[61] Rondanelli M., Cereda E., Klersy C., Faliva M.A., Peroni G., Nichetti M., Gasparri C., Iannello G., Spadaccini D., Infantino V. (2020). Improving Rehabilitation in Sarcopenia: A Randomized-Controlled Trial Utilizing a Muscle-Targeted Food for Special Medical Purposes. J. Cachexia. Sarcopenia Muscle.

[62] Bauer J.M., Verlaan S., Bautmans I., Brandt K., Donini L.M., Maggio M., McMurdo M.E.T., Mets T., Seal C., Wijers S.L. (2015). Effects of a Vitamin D and Leucine-Enriched Whey Protein Nutritional Supplement on Measures of Sarcopenia in Older Adults, the PROVIDE Study: A Randomized, Double-Blind, Placebo-Controlled Trial. J. Am. Med. Dir. Assoc..

[63] Yamada M., Kimura Y., Ishiyama D., Nishio N., Otobe Y., Tanaka T., Ohji S., Koyama S., Sato A., Suzuki M. (2019). Synergistic Effect of Bodyweight Resistance Exercise and Protein Supplementation on Skeletal Muscle in Sarcopenic or Dynapenic Older Adults. Geriatr. Gerontol. Int..

[64] Ekinci O., Yanlk S., Terzioǧlu Bebitoǧlu B., Yllmaz Akyüz E., Dokuyucu A., Erdem Ş. (2016). Effect of Calcium β-Hydroxy-β-Methylbutyrate (CaHMB), Vitamin D, and Protein Supplementation on Postoperative Immobilization in Malnourished Older Adult Patients with Hip Fracture. Nutr. Clin. Pract..

[65] Buondonno I., Sassi F., Carignano G., Dutto F., Ferreri C., Pili F.G., Massaia M., Nisoli E., Ruocco C., Porrino P. (2020). From Mitochondria to Healthy Aging: The Role of Branched-Chain Amino Acids Treatment: MATeR a Randomized Study. Clin. Nutr..

[66] VanDerVeer S., Markert R., Bickford B., Yuhas J., Pikman P., Wall T., Burtson K. (2021). Increasing Exercise Adherence among Elderly Patients with Chronic Disease in Primary Care: A Prospective Cohort Study. BMC Geriatr..

[67] Bauer J., Biolo G., Cederholm T., Cesari M., Cruz-Jentoft A.J., Morley J.E., Phillips S., Sieber C., Stehle P., Teta D. (2013). Evidence-Based Recommendations for Optimal Dietary Protein Intake in Older People: A Position Paper from the Prot-Age Study Group. J. Am. Med. Dir. Assoc..

[68] Pennings B., Groen B., de Lange A., Gijsen A.P., Zorenc A.H., Senden J.M.G., van Loon L.J.C. (2012). Amino Acid Absorption and Subsequent Muscle Protein Accretion Following Graded Intakes of Whey Protein in Elderly Men. Am. J. Physiol. Endocrinol. Metab..

[69] Wolfe R.R. (2017). Branched-Chain Amino Acids and Muscle Protein Synthesis in Humans: Myth or Reality?. J. Int. Soc. Sports Nutr..

[70] Wilkinson D.J., Hossain T., Hill D.S., Phillips B.E., Crossland H., Williams J., Loughna P., Churchward-Venne T.A., Breen L., Phillips S.M. (2013). Effects of Leucine and Its Metabolite β-Hydroxy-β-Methylbutyrate on Human Skeletal Muscle Protein Metabolism. J. Physiol..

[71] Gielen E., Beckwée D., Delaere A., De Breucker S., Vandewoude M., Bautmans I., Bautmans I., Beaudart C., Beckwée D., Beyer I. (2021). Nutritional Interventions to Improve Muscle Mass, Muscle Strength, and Physical Performance in Older People: An Umbrella Review of Systematic Reviews and Meta-Analyses. Nutr. Rev..

[72] Chang M.C., Choo Y.J. (2023). Effects of Whey Protein, Leucine, and Vitamin D Supplementation in Patients with Sarcopenia: A Systematic Review and Meta-Analysis. Nutrients.

[73] Rondanelli M., Gasparri C., Barrile G.C., Battaglia S., Cavioni A., Giusti R., Mansueto F., Moroni A., Nannipieri F., Patelli Z. (2022). Effectiveness of a Novel Food Composed of Leucine, Omega-3 Fatty Acids and Probiotic Lactobacillus Paracasei PS23 for the Treatment of Sarcopenia in Elderly Subjects: A 2-Month Randomized Double-Blind Placebo-Controlled Trial. Nutrients.

[74] Dzik K.P., Kaczor J.J. (2019). Mechanisms of Vitamin D on Skeletal Muscle Function: Oxidative Stress, Energy Metabolism and Anabolic State. Eur. J. Appl. Physiol..

[75] Beaudart C., Dawson A., Shaw S.C., Harvey N.C., Kanis J.A., Binkley N., Reginster J.Y., Chapurlat R., Chan D.C., Bruyère O. (2017). Nutrition and Physical Activity in the Prevention and Treatment of Sarcopenia: Systematic Review. Osteoporos. Int..

[76] Chevalley T., Brandi M.L., Cashman K.D., Cavalier E., Harvey N.C., Maggi S., Cooper C., Al-Daghri N., Bock O., Bruyère O. (2022). Role of Vitamin D Supplementation in the Management of Musculoskeletal Diseases: Update from an European Society of Clinical and Economical Aspects of Osteoporosis, Osteoarthritis and Musculoskeletal Diseases (ESCEO) Working Group. Aging Clin. Exp. Res..

[77] Mantuano P., Boccanegra B., Bianchini G., Conte E., De Bellis M., Sanarica F., Camerino G.M., Pierno S., Cappellari O., Allegretti M. (2021). BCAAs and Di-Alanine Supplementation in the Prevention of Skeletal Muscle Atrophy: Preclinical Evaluation in a Murine Model of Hind Limb Unloading. Pharmacol. Res..

[78] Baum J.I., Kim I.Y., Wolfe R.R. (2016). Protein Consumption and the Elderly: What Is the Optimal Level of Intake?. Nutrients.

